# Supplementary material for: Mutations in PCYT2 disrupt etherlipid biosynthesis and cause a complex hereditary spastic paraplegia
Source: Brain. 2019 Oct 22;142(11):3382–97. doi: 10.1093/brain/awz291 (PMC6821184; doi:10.1093/brain/awz291)
Supplement: awz291_Supplementary_Data [file awz291_supplementary_data.zip › awz291-Suppl_data/Supplementary_Data4.zip!/Supplementary information 3 - fibroblast lipidomics.zip!/Fibroblast lipidomics/Patient1 vs Patient2/Statistical plots and data/LPE[O] species/Random forest.pdf]

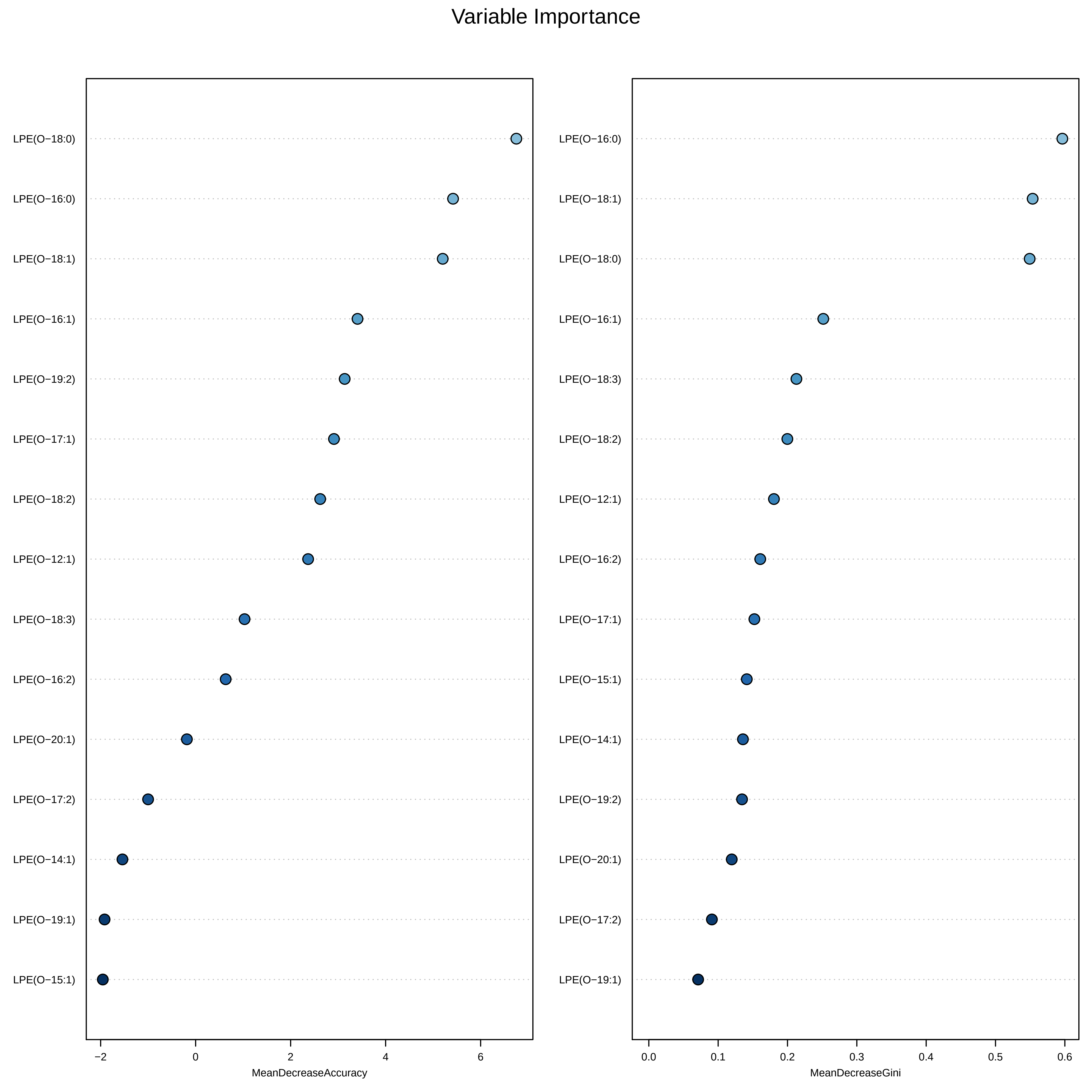
Variable Importance
LPE(O−18:0)
LPE(O−16:0)
LPE(O−18:1)
LPE(O−16:1)
LPE(O−19:2)
LPE(O−17:1)
LPE(O−18:2)
LPE(O−12:1)
LPE(O−18:3)
LPE(O−16:2)
LPE(O−20:1)
LPE(O−17:2)
LPE(O−14:1)
LPE(O−19:1)
LPE(O−15:1)
−2
0
2
4
6
MeanDecreaseAccuracy
LPE(O−16:0)
LPE(O−18:1)
LPE(O−18:0)
LPE(O−16:1)
LPE(O−18:3)
LPE(O−18:2)
LPE(O−12:1)
LPE(O−16:2)
LPE(O−17:1)
LPE(O−15:1)
LPE(O−14:1)
LPE(O−19:2)
LPE(O−20:1)
LPE(O−17:2)
LPE(O−19:1)
0.0
0.1
0.2
0.3
0.4
0.5
0.6
MeanDecreaseGini
